# Fritz Müller – Darwin's scientific correspondent in Brazil for 17 years

**DOI:** 10.1590/1678-4685-GMB-2018-0061

**Published:** 2018-08-02

**Authors:** Klaus Hartfelder

**Affiliations:** 1 Faculdade de Medicina de Ribeirão Preto, Universidade de São Paulo, Ribeirão Preto, SP, Brazil

**Keywords:** Darwinian theory, Für Darwin, Evolutionary Developmental Biology, crustcean phylogeny, Müllerian mimicry

**Figure 1:**
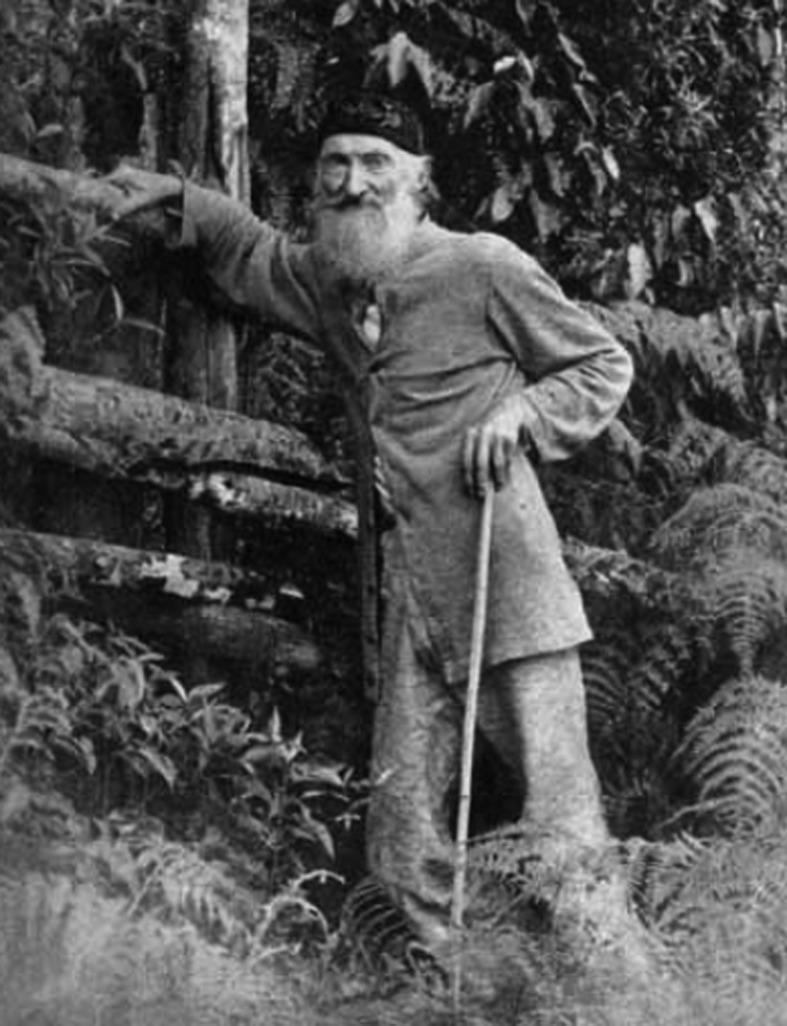
Fritz Müller (1822-1897). This is one of his best-known portraits, probably taken near his house in Blumenau.

Whenever I teach a class on Evolutionary Developmental Biology I show a photo of Charles Darwin together with one of Fritz Müller (Figure 1). Of course, all recognize Darwin, but only in most recent years one or two students mention having heard of Fritz Müller. Is this is the students fault? Certainly not, the problem is that his name and contribution to Darwinian theory rarely appear in Biology course curricula, not even in Brazil, where he lived and worked most of his life, but worldwide. In this respect, David West’s brilliantly written biography now gives a close view on this eminent scientific personality, and he takes us through the immense variety of topics that Fritz Müller worked on and studied during his life. Notably, based on careful observations from nature, Fritz Müller’s science was always guided by the purpose of putting to test Darwin’s theory of Natural Selection and Sexual Selection.

Johann Friedrich (Fritz) Theodor Müller, born 1822 in Thuringia, Germany, received his Doctorate in Zoology from the University of Berlin in 1844, having worked under the supervision of the famous anatomist and physiologist Johannes Peter Müller. He then went on to study medicine at the University of Greifswald, but driven by his critical spirit on religion and politics, in 1852 he decided to leave the ever more conservative Prussia and emigrated with his family and that of his brother Alfred to Santa Catarina, Brazil. There they joined the German settlement in Blumenau that had just been established by his family friend Hermann Blumenau. He built a house and cleared land in the Atlantic Forest to make a living as a settler, but gifted with a sharp eye and a trained biologist he also took to study the flora and fauna of the state Santa Catarina.

Although living far out in the woods, one might think that he would have been cut off from science, but this was not the case. He kept close contact, regularly exchanging letters with his other brother Hermann, also a well-known biologist, and many friends and colleagues active in biological sciences. He received regular shipments of books through his friend Max Schultze, later known for his Cell Theory and who became the Director of the Anatomical Institute of the University of Bonn. So, in 1861 he received the German translation of Darwin’s *On the Origin of Species*. He immediately recognized the immense value of this work and, by then temporarily employed as a teacher at the Provincial Lyceum (High School) in Desterro (now Florianopolis, the capital of Santa Catarina), he immediately set out to review and revise the studies that he had been conducting so far on crustaceans. During that time, his house was in an ideal location, right on the Atlantic coast, and between the years 1858 and 1865 he published nearly 30 papers on marine invertebrates, most of them in German, in the *Archiv für Naturgeschichte*. His main interest by then was to reconstruct a natural phylogenetic classification of the Crustacea, and he found this to be possible by exploring the progression of the larval stages, starting from the Nauplius. This view of bringing together Natural History and Evolution with and through Embryology (or what we would now call Developmental Biology) made Scott Gilbert refer to Fritz Müller as a “precursor of evolutionary developmental biology in the Americas” (Gilbert, 2008), a view also held by Marcellini *et al.* (2017) in their review on the past and present Evo-Devo research done in Latin America.

The plan for an embryology-based genealogy of Crustacea led Fritz Müller to write what is since considered a masterpiece of Darwinian theory, his book *Für Darwin* (Müller, 1864), copies of which his publisher sent to major authorities in the field, including Darwin. Being himself profoundly interested in the evolutionary history of crustaceans, Darwin wrote an enthusiastic letter to Fritz Müller and sponsored the translation of *Für Darwin* into English, with 1,000 copies published in 1869 under the title *Facts and Arguments for Darwin,* which included six additional comments by Fritz Müller. The title reflects exactly what Fritz Müller had in mind, and which is implicit in the German title. Nonetheless, this aspect was occasionally overlooked in translations to other languages, as the German word *Für* has a double meaning, it can be used both in a dedicatory sense, as well as in the sense ‘in favor of’. *Für Darwin* received several favorable reviews and was soon translated into French, Russian and other languages. A Portuguese translation, based on the English version, however had to wait until 1996, with a first translation based on the German original published in 2009 and a revised edition now in 2017 (Fontes and Hagen, 2017).

Clearly, *Für Darwin* is no easy reading for non-specialists, but its line of argumentation laid the ground for many subsequent historical landmarks in evolutionary biology, including Ernst Haeckel’s Biogenetic Law, which proposed that Ontogeny *recapitulates* Phylogeny. While Müller and Haeckel started to correspond in 1865, and did so over decades, Müller kept a critical distance to Haeckel’s recapitulation concept because, as a keen observer and from his ample knowledge on animal life histories, he realized that metamorphosis can be simplified and ‘falsified’ (*gefälscht* as Müller said) by natural selection, and that free-living larval stages can disappear in direct developers. It is through this critical perspective, that later was also profoundly expressed by Stephen Jay Gould in his *Ontogeny and Phylogeny* (Gould, 1977), whereby Müller and his contemporary Alexej Sewertzoff foresaw what is now conceived as the evolutionary impact of ontogeny on phylogeny, i.e., Evolutionary Developmental Biology.

To Darwin, Müller now became a main source for biological information for solving of what Darwin considered to be major problems for his theory, and hence, Fritz Müller’s name became the most cited reference from the fourth edition of the *Origin* onward. Although they never met in person, Darwin and Müller maintained a rich correspondence from 1865 until Darwin’s death in 1882, wherein Darwin continuously asked Müller for field observations on many topics that were of interest to him. Actually, it is worth remembering that the theory of Natural Selection was, at that time, much more readily accepted among German than by British scientists, and Müller was the most active naturalist among Darwin’s German correspondents, with a registry of 106 letters.

After his return from Desterro to Blumenau in 1867, Müller was appointed in 1876 to the position of a Traveling Naturalist to the Museum of Natural History in Rio de Janeiro and was now in a position to travel the unexplored forests and highlands of Santa Catarina, where he collected much valuable biological material. So in this respect, Darwin’s relationship with Müller was different from the one Darwin had with Henry Walter Bates and Alfred Russell Wallace, who, by that time, had already ended their extensive excursion activities to the Amazon and East Indies, respectively.

For instance, one of Darwin’s long-standing interests was to understand climbing plants, and he in fact sent his publication on climbing plants to Fritz Müller shortly after having received *Für Darwin*. Müller collected several climbers and described them in letters to Darwin, who readily excerpted these and sent them for publication to the *Journal of the Linnean Society*. This was Müller’s first publication in English. Similarly, Darwin’s book on orchid pollination by insects caught Müller’s interest and, being in an excellent position to collect orchids, he found a triandrous *Epidendrum* species on a Santa Catarina island, which he interpreted as a response to natural selection in consequence of a lack of pollinating insects.

Social insects were what Darwin considered another major problem for his theory, and he clearly expressed this in the *Origin.* In a letter, Müller reported several observations on termites and bees, and he clearly confirmed that the neuter caste of termites represents nymphs and not adults, as is the case in social Hymenoptera. Darwin compiled this information and, with Müller’s authorship, sent it as a communication to Nature, just like several subsequent excerpts from their correspondence.

Among the best know contributions of Fritz Müller to Biology are his observations and interpretations on mimicry - Müllerian mimicry as it is known since - and on chemical communication of insects. Mimicry in butterflies had been described before by Henry Walter Bates as a process whereby the wing pattern of a palatable species resembles that of an unpalatable one (Batesian mimicry), but Müller found similar forms of resemblance also between unpalatable species, so for him there was no way of distinguishing between model and copy. To explain this, Müller ingeniously came up with a mathematical model, whereby the advantage gained by two unpalatable species from their resemblance would be inversely related to the square of their individual numbers (Müller, 1879). Truly, this can be considered as one of the first mathematical models in Ecology and Evolution, and the paper originally published in German in *Kosmos* was immediately translated into English by Raphael Meldola, the Editor of the Proceedings of the Entomological Society of London. Evidently, Müller’s interpretation was criticized by several of his contemporaries because it implied that birds preying on insects would be capable of learning, but this is, of course, nothing that we would doubt nowadays.

Similarly, the notion that butterfly males may chemically communicate their presence to females came from field observations. Müller had read descriptions made by lepidopteran taxonomists on strange hair tufts (“sexual spots”) on the wings or abdomen of males, but not in females, and he came to associate these with chemical communication when he caught specimens in the wild and, when unfolding their wings, noted a strong smell coming from these tufts. This is what we now call sexual pheromone communication, and Michael Boppré, a renown chemical ecologist once considered: “*It is Müller who deserves the credit for being the pioneer in exploration of male scents in the Lepidoptera*” and also “*the first to distinguish between ‘sexual odours’ and ‘protective odours’ in butterflies*”.

Besides Darwin, with whom he corresponded until Darwin’s death in 1882, Müller had an extensive correspondence with other major thinkers and theorists of his time, such as the already mentioned Ernst Haeckel and August Weissmann, and actually it was Müller who convinced Weissmann to finally accept Francis Galton’s notion of the law of regression in the genetics of human hereditary stature, another pillar in Genetics.

Fritz Müller died in 1897 in Blumenau. In his obituary, Ernst Krause, one of Fritz Müller’s closest German friends and correspondents, mentioned that in a letter Darwin had referred to Fritz Müller as ‘the Prince of observers’. Another obituary appeared the same year in *Nature*.

Fritz Müller’s extensive scientific production - 263 articles and the book *Für Darwin* - and much of the scientific and private correspondence was later (1915-1921) compiled by his younger cousin Alfred Möller and published in three volumes. This collection laid the foundation for David A. West’s biography, but different from that very extensive compilation, West gives us a close portrait of the person Fritz Müller, of his contributions as scientist and also his private life, and he puts Müller’s science in context with that of his time. Actually this is his second book on Fritz Müller, the first one published in 2003 (West, 2003). Of course, I especially recommend the book to Brazilian biologists, not only because Fritz Müller considered himself Brazilian – he frequently signed as Frederico Müller – but also to show how important his Brazilian heritage was to the shaping of Darwinian theory and Evolutionary Developmental Biology. Finalizing, a beautifully illustrated bilingual biography edited by Westerkamp (2012) was published as an e-book in commemoration of Fritz Müller’s 190^th^ birthday, and it is now only four years until his bicentenary.
